# Characterization of a new anellovirus species infecting an ocelot (*Leopardus pardalis*) in Brazil

**DOI:** 10.1590/1678-4685-GMB-2023-0015

**Published:** 2023-12-04

**Authors:** Liliane T. F. Cavalcante, Matheus A. C. Cosentino, Mirela D’arc, Filipe R. R. Moreira, Ricardo Mouta, Anderson M. Augusto, Fernando Troccoli, Marcelo A. Soares, André F. Santos

**Affiliations:** 1 Universidade Federal do Rio de Janeiro, Departamento de Genética, Ilha do Fundão, RJ, Brazil.; 2 Instituto Nacional de Câncer, Programa de Oncovirologia, Rio de Janeiro, RJ, Brazil.; 3 Fundação Rio-Zoo, Rio de Janeiro, RJ, Brazil.

**Keywords:** Virome, felid, Anelloviridae

## Abstract

A complete genome of the first anellovirus infecting the wild felid *Leopardus pardalis* (ocelot) and a partial genome were assembled and annotated through high-throughput sequencing protocols followed by Sanger sequencing validation. The full-length virus obtained comprises 2,003 bp, while the partial genome comprises 1,224 bp. Phylogenetic analysis grouped these two sequences in two distinct clusters related to previously described Felidae anelloviruses. The ORF1 of the partial genome was identified as a new species provisionally called *Torque teno ocelot virus*, with 53.6% identity with its sister lineage. The complete genome was inferred as a new representative of the *Torque teno felid virus 3* species, with 73.28% identity to the closest reference. This study expands known virus diversity and the host span of anelloviruses.

The *Anelloviridae* family represents non-enveloped single-stranded viruses with circular negative-sense DNA, whose genomes range from 1.6 to 3.9 kb. The group is classified in 30 genera and 155 species by the International Committee of Virus Taxonomy (Varsani et al., 2021). Anelloviruses are a group of commensal viruses that cause asymptomatic chronic infections. No pathology has been associated with them to date (Kaczorowska and van der Hoek, 2020). They are one of the major constituents of the human virome (Arze et al., 2021) and their known diversity drastically increased through virome investigations of non-model animals (Ng et al., 2011; de Souza et al., 2018; Weber et al., 2020; Kraberger et al., 2021; Cosentino et al., 2022). In wild-life felids, anelloviruses have been identified in pumas (*Puma concolor*), bobcats (*Lynx rufus*), Canada lynx (*Lynx canadensis*) and caracals (*Caracal caracal*) (Kraberger *et al.*, 2021). In the present study, we characterize two novel anelloviruses in a Brazilian felid, the ocelot (*Leopardus pardalis*). 

During an annual examination to verify the health status of the captive felids at Rio de Janeiro City Zoo (RIOZOO) - Brazil, a blood sample was collected from a nine-year-old female ocelot (*Leopardus pardalis*). This project was performed with the authorization number 64515 from the Brazilian Institute for the Environment and Renewable Natural Resources (IBAMA). Plasma was filtered, centrifuged and the supernatant was discarded, leaving the bottom 0.2 mL to digestion with several nucleases. Viral nucleic acid was then extracted using QIAamp Mini Elute Virus Spin Kit (QIAGEN) and subjected to complementary DNA (cDNA) synthesis using Superscript III First-Strand Synthesis Supermix Kit (ThermoFisher). The second strand cDNA synthesis was performed with Klenow Fragment 3’-->5’ exo- (NEB). To get a highly efficient purification with superior quality DNA without salt carryover, the *Agencourt AMPure XP Kit* (Sinapse Biotecnologia) was used. To quantify double-strand DNA (dsDNA), the QuBit dsDNA High Sensitivity Assay Kit (ThermoFisher) was used, and the library was prepared using the Nextera XT DNA Library Preparation Kit (Illumina). Next-generation sequencing was conducted using the Illumina MiSeq V2 500-cycle kit. FASTq reads were processed with an in-house pipeline that included quality trimming, host genome filtering (*Felis catus* - #GCF_000181335.3, *Panthera tigris* - #GCA_000464555.1 and *Panthera pardus* - #GCA_001857705.1) and two rounds of BLASTx against RefSeq virus and nr (non-redundant) NCBI databases, as described by (D’arc et al., 2020). 

A total of 1,562 reads were assigned to the *Anelloviridae* family. These reads were *de novo* assembled using Geneious v.11.1.2, and the two biggest contigs were then submitted to BLASTn and two different anellovirus reference genomes were retrieved as best hits. A consensus sequence was used to design specific PCR primers to obtain the complete genome using genomic DNA from blood. Novel sequences were evolutionarily contextualized with a comprehensive *Anelloviridae* ORF1 dataset combining a dataset containing all known *Anelloviridae* genera (Cosentino et al., 2022) and one containing 220 novel sequences from multiple Felidae species (Kraberger et al., 2021). ORF1 genes were aligned by MAFFT v7.505 (Katoh and Standley, 2013) and TrimAL v.1.4 (Capella-Gutiérrez et al., 2009) was used to remove non informative regions with the “gappyout” option. A maximum likelihood phylogeny was inferred with IQ-TREE v.2.0.3 (Nguyen et al., 2015) and node support was estimated by 1,000 iterations to both metrics, the SH-like approximate likelihood ratio test (Guindon et al., 2010) and Ultrafast bootstrap (Minh et al., 2013). Tree visualization was obtained in R studio v.2022.12.0+353 with the package ggtree v.3.5.3 (Yu et al., 2017). Clustal Omega v.1.2.4 (Sievers et al., 2011) was used to calculate an identity matrix with the most closely related Felidae anellovirus sequences. Alignment and tree files can be found in Supplementary File S1.

A 2,003-bp full-length *Anellovirus*genome infecting *Leopardus pardalis* was assembled. This virus was provisionally named WF10 strain 1 (accession number: MK069470). Annotation was performed by aligning each ORF of the *Torque teno felis virus 2* (accession number: NC_038349) against the novel genome, which allowed the inference of homologous genome regions. Three open reading frames (ORF) encoding ORF 1 (1,224-bp), ORF 2 (321-bp) and ORF 3 (468-bp) were inferred. One partial genome was also assembled, comprising an incomplete ORF1 sequence with 1,173 nucleotides and was provisionally named WF10 strain 2 (accession number: OL449682). Felidae anelloviruses can be found in two major lineages and phylogenetic analysis of ORF1 gene grouped both new strains within feline anellovirus Group 1, in distinct lineages (Figure 1A). The WF10 strain 1 (accession number: MK069470) was inferred as a sister lineage to *Torque teno felis virus* (MT538137) (SH-aLRT = 100 / Bootstrap = 100). Sequences shared 73.8% of pairwise identity, confirming they belong to the *Torque teno felid virus 3* species in accordance with findings in Kraberger et al. (2021). It is worth noting the great genetic divergence observed within *Torque teno felid virus 3*, indicating that more than one viral species might be found in the present lineage upon further sampling (Supplementary File S1) (Figure 1B). 

**Figure 1 -:**
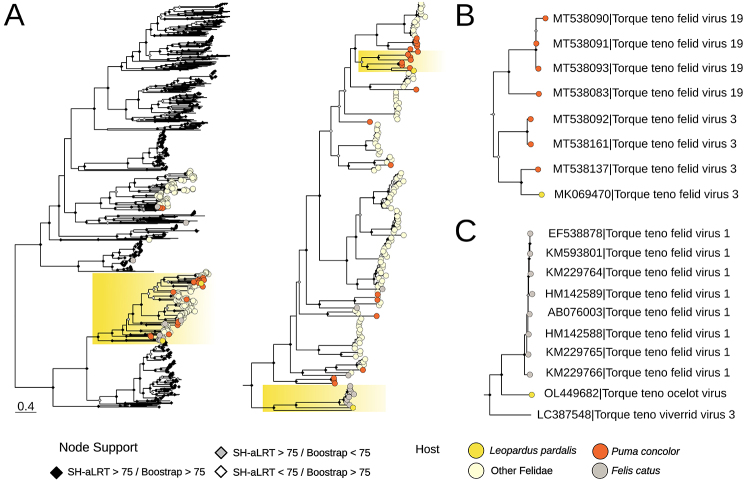
Maximum likelihood phylogeny of the ORF1 gene of the *Anelloviridae* family. Phylogeny of the ORF1 gene, inferred with a dataset of an alignment of 1183 aa length and 884 sequences under the LG+F+G4 model. The tree was midpoint-rooted for visualization purposes. Node shapes colored in black represent node support for SH-aLRT and Ultrafast Bootstrap equal or superior to 75. When only SH-aLRT was superior to this threshold, node shapes were represented in gray. Conversely, when only Ultrafast Bootstrap was superior, the white color was used. Nodes with support inferior to 75 in both parameters were not marked. Tip labels are colored according to the identified host, where orange tip labels represent anelloviruses identified in *Puma concolor*, dark yellow in *Leopardus pardalis*, gray in *Felis catus* and light yellow in other Felidae. (A) Maximum likelihood phylogeny of the ORF1 gene of the *Anelloviridae* family, with clades highlighted in yellow to evidence *Leopardus pardalis* anellovirus. (B) Monophyletic clade of *Torque teno felid virus 3* genomes identified in *L. pardalis*. (C) Monophyletic clade of the novel putative species *Torque teno ocelot virus*.

The ORF1 of the WF10 strain 2 (OL449682) was retrieved as sister to the *Torque teno felid virus 1* species clade (EF538878 - KM229766) with robust node support (SH-aLRT = 98 / Bootstrap = 100) (Figure 1C). As an outgroup, *Torque teno viverrid virus 3* (LC387548) was recovered with high support (SH-aLRT = 98.3 / Bootstrap = 100), indicating that the present monophyletic lineage likely descends from an ancient virus that co-diverged along the Suborder Feliformia. Based on ICTV species delimitation criteria (identity below 69%) (Varsani et al., 2021), the partial viral genome denoted a new species - provisionally named *Torque teno ocelot virus* - with 53.72% identity to the closest reference (accession number: EF538878). 

The captive *L. pardalis* explored by HTS protocols was coinfected by two different species of Anelloviruses, one new strain of *Torque teno felid virus 3* (accession number: MK069470) and a putative novel species provisionally named *Torque teno ocelot virus* (accession number: OL449682). *Anelloviridae* co-infection by multiple unique lineages is common in healthy humans (Arze et al., 2021), and this phenomenon was identified in felids as well (Kraberger et al., 2021). The characterization of these new viral species and sequences in a non-model animal expands *Anelloviridae* host span and may assist further studies aiming to characterize the complex evolutionary history of anelloviruses (Kraberger *et al.*, 2021; Cosentino et al., 2022). 

## Supplementary material

The following online material is available for this article:

File S1 - The tree file with maximum likelihood phylogeny inferred to contextualize the new viruses in ocelot and the fasta files to corresponding nucleotide and amino acid sequence alignment can be found in https://github.com/matheus-cosentino/2023_Novel-anelloviruses-in-ocelot.
